# Supplementary material for: Unravelling the non-classicality role in Gaussian heat engines
Source: Sci Rep. 2022 Jun 21;12:10412. doi: 10.1038/s41598-022-13811-z (PMC9213435; doi:10.1038/s41598-022-13811-z)
Supplement: Supplementary file 1 — Supplementary Information. [file 41598_2022_13811_MOESM1_ESM.pdf]

Supplementary Material to Unravelling the non-classicality role in Gaussian heat engines

A. de Oliveira Junior[1,2] and Marcos César de Oliveira*[3,4]

[1]*Faculty of Physics, Astronomy and Applied Computer Science, Jagiellonian University, 30-348 Kraków, Poland*
[2]*alexssandre.oliveirajunior@uj.edu.pl*
[3]*Instituto de Física Gleb Wataghin, Universidade Estadual de Campinas, 13083-859, Campinas, SP, Brazil*
[4]*marcos@ifi.unicamp.br*

(Dated: May 23, 2022)

I. P-REPRESENTABLE POSITIVE GAUSSIAN OPERATORS

This section derives the necessary and sufficient condition for the P-representability of a single bosonic mode. We begin by recalling that the characteristic function of a random variable ultimately determines its probability distribution. If a random variable admits a probability density function, then the characteristic function is its Fourier transform. The characteristic function χ_W of a Gaussian state can be recast in terms of its covariance matrix $\mathbf{V}$ as [1–3]

$$\chi_W(\mathbf{a}) = e^{-\frac{1}{2}\mathbf{a}^\dagger \mathbf{V}\mathbf{a}} \tag{1}$$

where $\mathbf{a} = (\alpha, \alpha^*)^\mathsf{T}$, and α is the complex eigenvalue of the annihilation operator a, whose eigenstate is the coherent state $|a\rangle$.

A positive Gaussian operator is said to be P-representable if it can be written as a mixture of coherent states with a proper probability distribution function. Otherwise, the state is not P-representable and, therefore, non-classical [4, 5]. The discrimination on whether a given state is classical or not is not an easy task. However, for Gaussian states, a specific criteria can be derived [3]. For this purpose, let us rewrite the P-function in terms of the parametrisation that we have been discussing,

$$P(\mathbf{a}) = \int d\mathbf{a}'\, e^{-\mathbf{a}^\dagger \sigma_z \mathbf{a}'} \chi(\mathbf{a}')\,, \tag{2}$$

where σ_z is the z-Pauli matrix, and $\chi_N(\mathbf{a})$ is the normally-ordered characteristic function that can be rewritten as

$$\chi_N(\mathbf{a}) = e^{\frac{1}{4}\mathbf{a}^\dagger\mathbf{a}}\chi_W(\mathbf{a}) = e^{\frac{1}{4}\mathbf{a}^\dagger\mathbf{a}} e^{-\frac{1}{2}\mathbf{a}^\dagger\mathbf{V}\mathbf{a}} = e^{\frac{1}{2}\mathbf{a}^\dagger\left(\frac{1}{2}\mathbb{1}-\mathbf{V}\right)\mathbf{a}}\,. \tag{3}$$

Substituting Eq.(3) into (2), and using the fact that $-\mathbf{a}^\dagger\sigma_z\mathbf{a}' = -\mathbf{a}^{\dagger\prime}\sigma_z\mathbf{a}$ [3], the P-function can be written as

$$P(\mathbf{a}) = \int d\mathbf{a}'\, e^{\mathbf{a}^{\dagger\prime}\left(\mathbf{V}-\frac{1}{2}\right)\mathbf{a}'} = \frac{1}{\sqrt{\det\left(\mathbf{V}-\frac{1}{2}\right)}} e^{\frac{1}{2}(\sigma_z\mathbf{a})^\mathsf{T}\sigma_x\left(\mathbf{V}-\frac{1}{2}\right)^{-1}(\sigma_z\mathbf{a})}\,, \tag{4}$$

where σ_x is the x-Pauli matrix. It can be verified that $(\sigma_z\mathbf{a})^\mathsf{T} = \mathbf{a}\sigma_z$ and $\mathbf{a}\sigma_z\mathbf{T} = -\mathbf{a}^\dagger\sigma_z$, thus

$$P(\mathbf{a}) = \frac{1}{\sqrt{\det\left(\mathbf{V}-\frac{1}{2}\right)}} e^{-\frac{1}{2}\mathbf{a}^\dagger\sigma_z\left(\mathbf{V}-\frac{1}{2}\right)\sigma_z\mathbf{a}} \equiv \sqrt{\det(\mathbf{P})}\, e^{-\frac{1}{2}\mathbf{a}^\dagger\mathbf{P}\mathbf{a}}\,, \tag{5}$$

where

$$\mathbf{P} = \sigma_z\left(\mathbf{V}-\frac{\mathbb{1}}{2}\right)^{-1}\sigma_z\,, \tag{6}$$

From this result we may conclude that, for a single-mode Gaussian state described by a covariance matrix $\mathbf{V}$ is said to be P-representable if

$$\bar{n} > |m|\,, \tag{7}$$

Note that a P-representable state, $\mathbf{P}$ must be non-negative, which means that $(\mathbf{V} - \mathbb{1}/2) \geq 0$. This condition requires eigenvalues greater than or equal to zero, or alternatively, a non-negative determinant.

$$\det\left(\mathbf{V}-\frac{\mathbb{I}}{2}\right) = \begin{vmatrix} \bar{n} & m \\ m^* & \bar{n} \end{vmatrix} = \bar{n}^2 - |m|^2 \geq 0\,. \tag{8}$$

It is important to emphasise that $\bar{n}$ is not necessarily the mean number of thermal photons $\bar{n}_{\text{th}}$, but the mean number of photons corresponds to the distribution in which the system is described.

[1] M. Scully and M. Zubairy, *Quantum Optics* (Cambridge University Press, 1997).
[2] C. Gerry, P. Knight, and P. Knight, *Introductory Quantum Optics* (Cambridge University Press, 2005).
[3] B.-G. Englert and K. Wódkiewicz, International Journal of Quantum Information **01**, 153 (2003), https://doi.org/10.1142/S0219749903000206.
[4] R. J. Glauber, Phys. Rev. **131**, 2766 (1963).
[5] E. C. G. Sudarshan, Phys. Rev. Lett. **10**, 277 (1963).
